# Prediction of Ascending Aortic Dilation and Analysis of Influencing Factors in Bicuspid Aortic Valve Patients Using an Explainable Machine Learning Model

**DOI:** 10.1155/crp/9933498

**Published:** 2026-06-13

**Authors:** Sijing He, Ning Yan, Guixuan Nie, Fang Wang, Xingyue Yang, Lili Wang, Lisha Na

**Affiliations:** ^1^ First Clinical Medical College, Ningxia Medical University, Yinchuan, 75004, China, nxmu.edu.cn; ^2^ Department of Cardiac Function Examination of Heart Centre, General Hospital of Ningxia Medical University, Yinchuan, 75004, China, nxmu.edu.cn; ^3^ Department of Cardiac Function Examination, Cardiovascular and Cerebrovascular Disease Hospital, General Hospital of Ningxia Medical University, Yinchuan, China, nxmu.edu.cn

**Keywords:** ascending aortic dilation, bicuspid aortic valve, machine learning, matrix metalloproteinase, SHAP

## Abstract

**Objective:**

Developing a machine learning (ML) model to predict the risk of ascending aortic dilation in patients with a bicuspid aortic valve (BAV). Using SHapley Additive exPlanations (SHAP) to interpret and visualize the model.

**Methods:**

This study enrolled 102 BAV patients, who were divided into two subgroups based on ascending aorta diameter (dilated and nondilated). All participants underwent routine echocardiography, clinical baseline data collection, and measurement of plasma matrix metalloproteinases (MMPs) and their tissue inhibitors (TIMPs). Feature selection was performed using univariate analysis followed by the least absolute shrinkage and selection operator (LASSO)–logistic regression (LR) method. Five common ML prediction models were developed: support vector machine (SVM), LR, gradient‐boosting machine (GBM), neural network (NNET), and Naïve Bayes (NB) classifier. To identify the best‐performing predictive model for ascending aortic dilation in BAV patients, an evaluation of predictive efficacy was carried out by employing ROC curves, calibration curves, and DCA curves. Finally, the optimal model’s predictions were interpreted using SHAP.

**Results:**

Random allocation of the entire patient population resulted in a training set (*n* = 72) and a test set (*n* = 30). Application of LASSO‐LR analysis revealed age, plasma MMP‐2, and peak aortic valve velocity (Vmax AV) as independent factors influencing ascending aortic dilation in BAV patients. These predictors were integrated into the subsequent ML model. GBM model achieved the optimal overall performance after 5‐fold cross‐validation. It attained an AUC of 0.982 on the training set, alongside an AUC of 0.915 on the test set. Calibration curves and DCA curves further demonstrate that the model exhibits good calibration and clinical net benefit. According to SHAP analysis, elevated plasma MMP‐2 contributed the most to the GBM model’s predictions, followed by increased age and elevated Vmax AV.

**Conclusions:**

The GBM model offers a valuable tool for predicting ascending aortic dilation in BAV patients. Moreover, SHAP analysis enhances the model’s utility by providing clear, actionable insights for clinical management.

## 1. Introduction

Bicuspid aortic valve (BAV) represents the most common congenital cardiovascular disease, affecting nearly 2% in the general population [1]. BAV is fundamentally a progressive valvulo‐aortopathy. Aortic dilation (predominantly ascending aorta) occurs in more than half of BAV patients [2]. Compared to the general population, BAV patients face an eightfold increased risk of aortic dissection, which significantly contributes to their overall mortality rate [3, 4].

The progression of BAV aortopathy is often silent, and its pathogenesis involves both genetic and hemodynamic factors [5]. These challenges have resulted in a lack of targeted clinical strategies for preventing and monitoring the disease [6]. In clinical practice, echocardiography is the preferred imaging modality for screening, diagnosing, and monitoring BAV aortopathy [7]. Despite the fact that aortic diameter remains the sole surgical indication for aortopathy associated with BAV, numerous studies have demonstrated that aortic dissection can occur in BAV patients even when the aortic dimensions fall below the threshold for surgical intervention [8, 9].

Therefore, there is an urgent clinical need to conduct prospective studies to explore circulating biomarkers associated with BAV aortopathy, with the aim of clarifying their pathophysiological mechanisms for early identification of structural changes in the aortic wall. Previous studies have confirmed that heightened matrix metalloproteinases (MMPs) activity and an imbalance between MMPs with tissue inhibitors of matrix metalloproteinases (TIMPs) are critical markers of impaired medial layer remodeling in BAV patients′ aortas [10, 11].

Recently, machine learning (ML) algorithms have proliferated and been successfully adopted within the field of medicine [12, 13]. Unlike traditional logistic regression (LR), ML models can capture complex, nonlinear relationships within data, often yielding superior predictive performance. Nevertheless, the “black‐box” character of ML algorithms, resulting from their complex logic and lack of interpretability, constitutes a barrier to their widespread clinical adoption [14]. This study integrated multidisciplinary data, including circulating biomarkers (plasma MMPs and TIMPs), echocardiographic parameters, and clinical baseline characteristics to identify risk factors and develop an optimal predictive mode for ascending aortic dilatation in BAV patients. Furthermore, to enhance interpretability, we employed SHapley Additive exPlanations (SHAP) analysis to provide visual explanations of the optimal model’s predictions. The ultimate goal is to establish a clinically applicable tool that provides a scientific basis for decision‐making in BAV aortopathy.

## 2. Methods

### 2.1. Study Population

During the period from June 2024 to June 2025, 102 consecutive adult patients diagnosed with BAV were enrolled. The diagnostic criterion for BAV was the identification of an aortic valve with two leaflets exhibiting a “fish mouth” opening pattern on left parasternal short‐axis views by transthoracic echocardiography (TTE) [15]. Valve phenotypes were subsequently classified according to the number and orientation of the raphe. Patients were excluded due to these criteria: (1) history of hypertension, diabetes, dyslipidemia, or other cardiovascular diseases; (2) prior cardiac or major vascular surgery; (3) presence of more than mild aortic stenosis or regurgitation; (4) presence of more than mild stenosis or regurgitation of the mitral or tricuspid valve; and (5) poor image quality precluding adequate analysis. Approval for this study was obtained from the Medical Ethics Committee of General Hospital of Ningxia Medical University (approval no. KYLL‐2024‐1696). All enrolled participants signed written informed consent in line with the tenets of the Declaration of Helsinki. Additionally, the following baseline data were obtained from all participants: age, sex, height, body weight, blood pressure, body surface area (BSA), body mass index (BMI), smoking history, triglycerides (TG), total cholesterol (TC), high‐density lipoprotein cholesterol (HDL‐C), low‐density lipoprotein cholesterol (LDL‐C), and fasting blood glucose (GLU).

### 2.2. Echocardiography

All patients were examined by TTE via GE Vivid E95 ultrasound system (Horten, Norway) with an M5S (1.7–3.3 MHz) transducer. All measurements were analyzed by two experienced sonographers in line with current guidelines [16], with each parameter ultimately calculated as the average of three measurements.

Left ventricular ejection fraction (LVEF) and left atrial volume index (LAVI) were determined via the biplane Simpson’s method. The left ventricular mass index (LVMI) was computed using its standard formula. Hemodynamic evaluation of the aortic valve encompassed recording the peak velocity (Vmax AV) and mean pressure gradient (MPG), which were acquired by continuous‐wave Doppler from the apical five‐chamber view. Assessment of left ventricular diastolic function employed pulsed‐wave and tissue Doppler imaging in the apical four‐chamber view. At the mitral orifice, the peak early (E) and late (A) diastolic filling velocities were recorded to determine the E/A ratio. Furthermore, the peak early diastolic mitral annular velocities (e′) at the septal and lateral walls were obtained, enabling the calculation of the average E/e′ ratio.

The proximal ascending aorta diameter was measured 2 cm above the sinotubular junction in the long‐axis view, and the ascending aortic diameter index was calculated. Based on this diameter, BAV patients were categorized into two groups according to established criteria [17, 18]: a dilated ascending aorta group (> 35 mm, *n* = 53) and a nondilated ascending aorta group (≤ 35 mm, *n* = 49).

### 2.3. Laboratory Examinations

All study subjects underwent fasting blood collection into tubes with ethylenediaminetetraacetic acid (EDTA) anticoagulant. Plasma, separated by centrifugation (3000 rpm, 15 min), was aliquoted and frozen at −80°C for subsequent analysis. Commercial enzyme‐linked immunosorbent assay (ELISA) kits from R&D Systems (Wuhan, China) were used to measure plasma levels of MMP‐1, MMP‐2, MMP‐3, and MMP‐9 and TIMP‐1, TIMP‐2, and TIMP‐4. An ELISA kit from Boster Biological Technology (Wuhan, China) was employed for TIMP‐3. The respective MMP/TIMP ratios were then computed. All measurements were performed in triplicate according to the manufacturer’s protocols. Lipid parameters (TG, TC, HDL‐C, and LDL‐C) and fasting GLU were analyzed using the identical sample processing and storage protocol.

### 2.4. Statistical Analysis

Data analysis was conducted using R software (Version 4.3.3; R Foundation for Statistical Computing, Vienna, Austria). Continuous variables first underwent normality testing with the Shapiro–Wilk method. Data conforming to a normal distribution are reported as mean ± standard deviation and underwent group comparisons via the independent samples *t-*test. In contrast, non‐normally distributed data are summarized as median (interquartile range) and were compared via the Mann–Whitney *U* test. Categorical variables are expressed as frequency (percentage), and the chi‐square test was applied for their analysis. Statistical significance was defined by a two‐sided *p* value < 0.05.

A univariate analysis was first conducted to compare the total of 48 variables between patients stratified according to the status of ascending aortic dilation. Those variables showing a significant correlation with aortic dilation (*p* < 0.05) were further processed using the least absolute shrinkage and selection operator (LASSO) regression within the R package “glmnet.” Feature screening was enhanced by optimizing the tuning parameter *λ* via 10‐fold cross‐validation. Ultimately, the variables retained from the LASSO analysis were used in a multivariable LR to establish independent predictors of ascending aortic dilation.

### 2.5. Prediction Model Development and Evaluation

**FIGURE 1:**
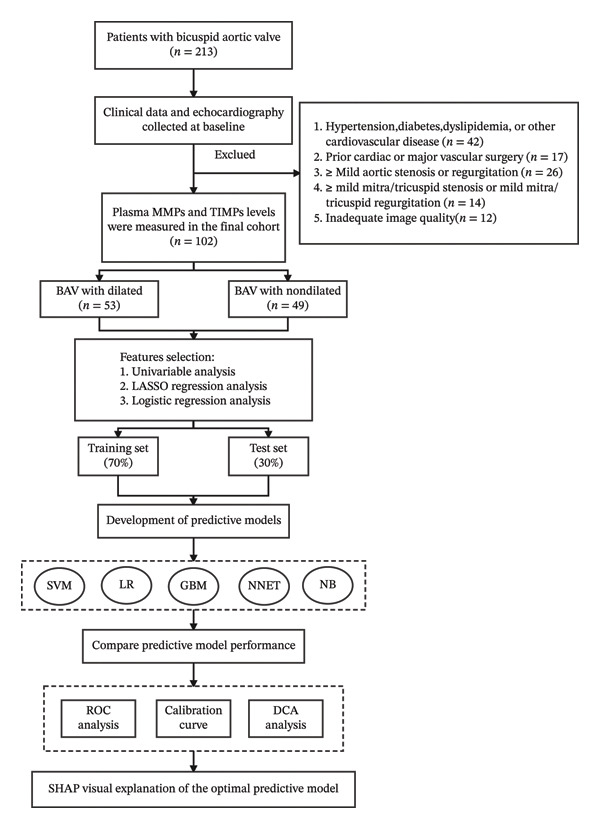
Flowchart. AUC, area under the curve; BAV, bicuspid aortic valve; DCA, decision curve analysis; GBM, gradient‐boosting machine; LASSO, least absolute shrinkage and selection operator; LR, logistic regression; MMPs, matrix metalloproteinases; NB, Naïve Bayes; NNET, neural network; SHAP, SHapley Additive exPlanations; SVM, support vector machine; TIMPs, tissue inhibitors of matrix metalloproteinases.

Random division of the study cohort, performed using the “caret” package in R, resulted in a training set (70%; *n* = 72) and a test set (30%; *n* = 30). The ML models developed in this study comprised support vector machine (SVM), LR, gradient‐boosting machine (GBM), neural network (NNET), and Naïve Bayes (NB). To evaluate the stability and generalization ability of the model, 5‐fold cross‐validation was applied. Additionally, the receiver operating characteristic (ROC) curve and the area under the curve (AUC) were utilized to assess the model’s discriminative performance. Meanwhile, the reliability of the model was evaluated based on metrics including accuracy, sensitivity, specificity, F1‐score, and Brier score. A comparison of AUC values between the models was performed using the DeLong test. Model calibration was assessed using calibration curves and the Hosmer–Lemeshow test, whereas clinical utility was determined by decision curve analysis (DCA). Finally, variable importance was quantified by applying SHAP to the optimal model, which also provided global and local interpretability through the calculation of contribution values. Figure 1 illustrates the overall study workflow.

## 3. Results

### 3.1. Baseline Characteristics

The study cohort, after screening against the criteria, consisted of 102 BAV patients. These individuals were categorized by the status of ascending aortic dilation into a group with dilation (*n* = 53) and a group without (*n* = 49). Patients with dilation were significantly older than those without (*p* < 0.001). Comparison of the remaining clinical parameters showed no significant intergroup disparities (all *p* > 0.05). All baseline data are summarized in Table 1.

**TABLE 1:** Baseline characteristics of BAV patients.

Variable	BAV with dilated (*N* = 53)	BAV with nondilated (*N* = 49)	*p* value
Age [years, M (Q1, Q3)]	45.00 (36.50, 52.00)	34.00 (27.00, 36.50)	< 0.001
Male [*n*, (%)]	37 (69.81%)	29 (59.18%)	0.262
Height (cm, mean ± SD)	1.69 ± 0.08	1.67 ± 0.07	0.268
Weight (kg, mean ± SD)	70.07 ± 14.07	67.95 ± 11.02	0.402
Body surface area (m^2^, mean ± SD)	1.80 ± 0.20	1.76 ± 0.17	0.353
Body mass index [kg/m^2^, M (Q1, Q3)]	23.67 (21.64, 25.62)	23.52 (21.95, 25.53)	0.979
Systolic blood pressure [mmHg, M (Q1, Q3)]	120.00 (111.50, 132.00)	121.00 (106.50, 129.00)	0.279
Diastolic blood pressure [mmHg, M (Q1, Q3)]	84.00 (76.00, 87.50)	81.00 (74.00, 86.00)	0.348
Smoking [*n*, (%)]	24 (45.28%)	15 (30.61%)	0.128
TG [mmol/L, M (Q1, Q3)]	1.54 (1.11, 3.51)	1.42 (1.02, 2.92)	0.789
TC [mmol/L, M (Q1, Q3)]	3.72 (3.06, 4.18)	3.43 (2.87, 4.07)	0.320
HDL‐C [mmol/L, M (Q1, Q3)]	1.11 (0.99, 1.34)	1.07 (0.87, 1.29)	0.209
LDL‐C [mmol/L, M (Q1, Q3)]	2.37 (2.12, 2.85)	2.27 (1.93, 2.78)	0.525
GLU (mmol/L, mean ± SD)	5.50 ± 0.60	5.62 ± 0.59	0.325

*Note:* GLU, blood glucose; TG, triglyceride.

Abbreviations: BAV, bicuspid aortic valve; HDL‐C, high‐density lipoprotein cholesterol; LDL‐C, low‐density lipoprotein cholesterol; TC, total cholesterol.

### 3.2. Echocardiographic Parameters

The dilated group exhibited significantly higher values for Vmax AV, MPG, ascending aortic diameter, and ascending aorta diameter index compared to the nondilated group (all *p* < 0.001). Conversely, parameters including LVEF, LVMI, E/A ratio, average E/e′, LAVI, and valve phenotype showed no statistically significant intergroup differences (all *p* > 0.05). Table 2 presents these data.

**TABLE 2:** Echocardiographic parameters of BAV patients.

Variable	BAV with dilated (*N* = 53)	BAV with nondilated (*N* = 49)	*p* value
LVEF (%, mean ± SD)	68.17 ± 4.62	67.97 ± 4.62	0.824
LVMI (g/m^2^, mean ± SD)	78.64 ± 13.85	74.39 ± 15.67	0.149
E/A (mean ± SD)	1.16 ± 0.33	1.21 ± 0.43	0.455
E/e′ average [M (Q1, Q3)]	6.41 (5.74, 7.75)	6.00 (5.14, 6.77)	0.063
LA volume index [mL/m^2^, M (Q1, Q3)]	24.60 (21.77, 29.31)	25.34 (20.56, 28.37)	0.476
Vmax AV [m/s, M (Q1, Q3)]	1.40 (1.26, 1.55)	1.20 (1.08, 1.33)	< 0.001
MPG [mmHg, M (Q1, Q3)]	4.34 (3.13, 5.42)	2.94 (2.49, 3.57)	< 0.001
Valve phenotype [*n*, (%)]			0.217^a^
R‐L fusion	27 (50.94%)	30 (61.22%)	
R‐N fusion	12 (22.64%)	8 (16.33%)	
L‐N fusion	9 (16.98%)	3 (6.12%)	
2‐Sinus	5 (9.43%)	8 (16.33%)	
AAo diameter [mm, M (Q1, Q3)]	38.00 (36.41, 41.17)	29.70 (27.60, 31.91)	< 0.001
AAo diameter index (cm/m^2^, mean ± SD)	2.19 ± 0.29	1.69 ± 0.21	< 0.001

*Note:* AAo: ascending aorta; E/A, ratio of the early to late ventricular filling velocities; E/e′, ratio between early mitral in flow velocity and mitral annular early diastolic velocity; Vmax AV, aortic valve peak velocity.

Abbreviations: BAV, bicuspid aortic valve; LV, left ventricular; MPG, mean pressure gradient.

^a^Fisher’s exact test was applied to calculate *p* values.

### 3.3. Plasma Levels of MMPs, TIMPs, and MMP/TIMP Ratios

BAV patients exhibiting aortic dilation displayed markedly elevated plasma concentrations of several biomarkers relative to those without dilation. Concentrations of MMP‐2 (*p* < 0.001), MMP‐3 (*p* = 0.013), and MMP‐9 (*p* = 0.028) were notably increased. This pattern of elevation extended to TIMP‐1 and TIMP‐2, along with specific MMP/TIMP ratios such as MMP‐2/TIMP‐1, MMP‐2/TIMP‐2, MMP‐2/TIMP‐3, and MMP‐2/TIMP‐4 (all *p* < 0.001), in addition to the MMP‐3/TIMP‐3 and MMP‐9/TIMP‐3 ratios (all *p* = 0.043). Table 3 contains the complete dataset.

**TABLE 3:** Plasma levels of MMPs, TIMPs, and MMP/TIMP ratios of BAV patients.

Variable	BAV with dilated (*N* = 53)	BAV with nondilated (*N* = 49)	*p* value
MMP‐1 [ng/mL, M (Q1, Q3)]	1.57 (0.98, 2.67)	1.40 (0.88, 2.25)	0.435
MMP‐2 [ng/mL, M (Q1, Q3)]	159.24 (123.12, 197.39)	94.82 (74.99, 132.90)	< 0.001
MMP‐3 [ng/mL, M (Q1, Q3)]	6.49 (4.47, 8.75)	4.86 (3.34, 7.11)	0.013
MMP‐9 [ng/mL, M (Q1, Q3)]	14.07 (9.03, 24.80)	9.33 (6.54, 16.94)	0.028
TIMP‐1 [ng/mL, M (Q1, Q3)]	61.21 (50.06, 74.30)	51.35 (40.32, 61.93)	< 0.001
TIMP‐2 [ng/mL, M (Q1, Q3)]	85.56 (73.67, 97.16)	66.19 (57.45, 78.77)	< 0.001
TIMP‐3 [ng/mL, M (Q1, Q3)]	2.30 (1.76, 3.40)	2.56 (1.77, 3.87)	0.562
TIMP‐4 [ng/mL, M (Q1, Q3)]	3.94 (3.42, 4.73)	3.49 (2.91, 4.30)	0.078
MMP‐1/TIMP‐1 [M (Q1, Q3)]	0.03 (0.02, 0.04)	0.03 (0.02, 0.05)	0.347
MMP‐1/TIMP‐2 [M (Q1, Q3)]	0.02 (0.01, 0.03)	0.02 (0.01, 0.03)	0.382
MMP‐1/TIMP‐3 [M (Q1, Q3)]	0.71 (0.32, 1.28)	0.48 (0.34, 1.01)	0.420
MMP‐1/TIMP‐4 [M (Q1, Q3)]	0.47 (0.23, 0.66)	0.45 (0.27, 0.75)	0.735
MMP‐2/TIMP‐1 [M (Q1, Q3)]	2.64 (2.07, 3.15)	2.01 (1.48, 2.45)	< 0.001
MMP‐2/TIMP‐2 [M (Q1, Q3)]	1.83 (1.51, 2.34)	1.51 (1.22, 1.79)	< 0.001
MMP‐2/TIMP‐3 [M (Q1, Q3)]	63.65 (38.91, 104.61)	36.37 (24.53, 57.50)	< 0.001
MMP‐2/TIMP‐4 (mean ± SD)	42.15 ± 14.33	28.95 ± 12.66	< 0.001
MMP‐3/TIMP‐1 [M (Q1, Q3)]	0.11 (0.08, 0.18)	0.09 (0.07, 0.15)	0.408
MMP‐3/TIMP‐2 [M (Q1, Q3)]	0.08 (0.05, 0.11)	0.07 (0.05, 0.12)	0.604
MMP‐3/TIMP‐3 [M (Q1, Q3)]	2.73 (1.47, 4.30)	1.80 (1.19, 3.17)	0.043
MMP‐3/TIMP‐4 [M (Q1, Q3)]	1.72 (1.04, 2.28)	1.31 (0.81, 2.21)	0.136
MMP‐9/TIMP‐1 [M (Q1, Q3)]	0.25 (0.16, 0.35)	0.20 (0.14, 0.27)	0.234
MMP‐9/TIMP‐2 [M (Q1, Q3)]	0.17 (0.12, 0.24)	0.16 (0.10, 0.22)	0.408
MMP‐9/TIMP‐3 [M (Q1, Q3)]	5.21 (3.36, 11.40)	3.67 (2.21, 8.16)	0.043
MMP‐9/TIMP‐4 [M (Q1, Q3)]	3.45 (2.16, 5.69)	3.00 (1.34, 5.72)	0.171

*Note:* MMPs, matrix metalloproteinases; TIMPs, tissue inhibitors of matrix metalloproteinases.

Abbreviation: BAV, bicuspid aortic valve.

### 3.4. Influencing Factors for Ascending Aortic Dilation in BAV Patients

**FIGURE 2:**
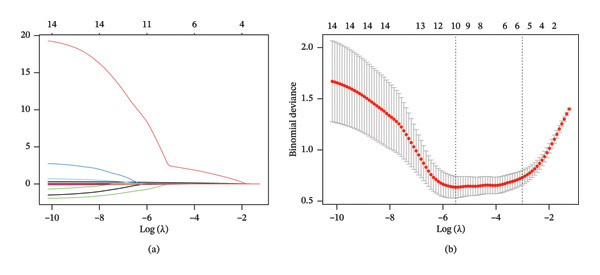
Screening process of predictive features by LASSO regression.

LASSO regression was applied to the 14 variables that showed univariate significance (*p* < 0.05). The optimal penalty term (*λ*) was determined using 10‐fold cross‐validation. Figure 2 illustrates that the number of selected features was progressively reduced until none remained. This process generated two key parameters: λ‐min (the value yielding the minimum cross‐validation error) and λ‐1se (the largest value with an error within one standard error of the minimum). Following the λ‐1se standard, the final selected features for the following LR were age, Vmax AV, MMP‐2, TIMP‐2, and the MMP‐2/TIMP‐3 ratio.

The results of the univariate and multivariate analyses are presented in Table 4. Univariate analysis identified age, Vmax AV, MMP‐2, TIMP‐2, and the MMP‐2/TIMP‐3 ratio as significant influencing factors for ascending aortic dilation in BAV patients. However, the multivariate analysis demonstrated that only age, Vmax AV, and MMP‐2 were independent influencing factors.

**TABLE 4:** Logistic regression analysis of factors associated with ascending aortic dilation in BAV patients.

Variable	Coefficient	*p* value	Odds ratio (95% CI)
*Univariate analysis*			
Age	0.107	< 0.001	1.113 (1.059–1.169)
Vmax AV	3.578	< 0.001	35.792 (5.327–240.494)
MMP‐2	0.041	< 0.001	1.042 (1.025–1.059)
TIMP‐2	0.067	< 0.001	1.069 (1.036–1.103)
MMP‐2/TIMP‐3	0.028	< 0.001	1.028 (1.013–1.044)

*Multivariate analysis*			
Age	0.181	< 0.001	1.198 (1.094–1.312)
Vmax AV	2.342	0.042	10.399 (1.087–99.496)
MMP‐2	0.052	< 0.001	1.054 (1.023–1.085)
TIMP‐2	0.053	0.051	1.054 (1.000–1.112)
MMP‐2/TIMP‐3	0.021	0.136	1.022 (0.993–1.051)

*Note:* MMPs, matrix metalloproteinases; TIMPs, metalloproteinases; Vmax AV, aortic valve peak velocity.

Abbreviation: BAV, bicuspid aortic valve.

### 3.5. Development and Comparison of Predictive Models

Comparison of the training (*n* = 72) and test (*n* = 30) cohorts, which were established by random allocation, revealed no statistically significant differences in any variables (all *p* > 0.05). Using the three independent factors for BAV with ascending aortic dilation identified by the LASSO‐LR analysis, we developed 5 ML models: SVM, LR, GBM, NNET, and NB. We employed a 5‐fold cross‐validation to assess and compare the predictive capabilities of these models.

**FIGURE 3:**
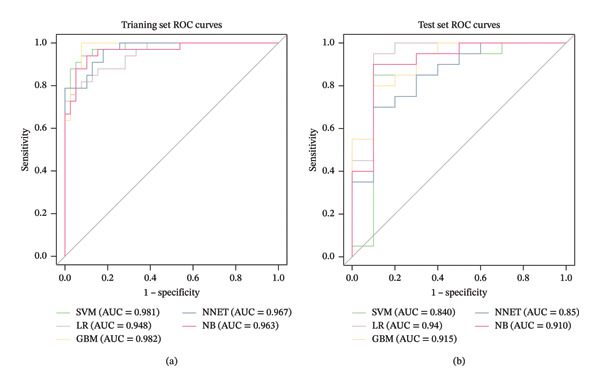
ROC curves of the predictive models. (a) Training set; (b) test set. The horizontal axis represents 1–specificity, and the vertical axis represents sensitivity. Curves in different colors correspond to different models, and AUC indicates the area under the curve.

Figure 3 shows the ROC curves and corresponding AUC values of the five models. Among the models evaluated on the training set, the discriminative ability of the GBM (AUC = 0.982) and SVM (AUC = 0.981) models was the highest, followed by NNET (AUC = 0.967), NB (AUC = 0.963), and LR (AUC = 0.948) in descending order. On the test set, the LR (AUC = 0.940) and GBM (AUC = 0.915) models demonstrated the best discriminative performance, followed by NB (AUC = 0.910), NNET (AUC = 0.850), and SVM (AUC = 0.840) in that order. Furthermore, Table 5 further details the other performance evaluation metrics for each model on both the training and test sets. On the training set, the SVM (0.931) and GBM (0.917) models achieved the highest accuracy. However, the GBM model demonstrated the most balanced sensitivity (0.909) and specificity (0.923), and its *F*1‐score (0.909) was second only to that of the SVM model (0.921). On the test set, the LR model (accuracy = 0.900, sensitivity = 0.900, specificity = 0.900, and *F*1‐score = 0.923) and the GBM model (accuracy = 0.833, sensitivity = 0.850, specificity = 0.800, and *F*1‐score = 0.872) demonstrated the best overall performance and the most balanced metrics. However, based on the Delong’s test, the difference in diagnostic performance between the LR and GBM models on the test set was not statistically significant (*p* = 0.629).

**TABLE 5:** Model performance and comparison.

Classifier	AUC	Accuracy	Sensitivity	Specificity	*F*1‐score	Brier score
*Training set*						
SVM	0.981	0.931	0.879	0.974	0.921	0.059
LR	0.948	0.875	0.818	0.923	0.857	0.095
GBM	0.982	0.917	0.909	0.923	0.909	0.053
NNET	0.967	0.861	0.788	0.923	0.839	0.084
NB	0.963	0.861	0.758	0.949	0.833	0.079

*Test set*						
SVM	0.840	0.867	0.850	0.900	0.895	0.126
LR	0.940	0.900	0.900	0.900	0.923	0.080
GBM	0.915	0.833	0.850	0.800	0.872	0.095
NNET	0.850	0.733	0.650	0.900	0.765	0.177
NB	0.910	0.800	0.750	0.900	0.833	0.131

*Note:* NNET, neural network.

Abbreviations: AUC, area under the curve; GBM, gradient‐boosting machine; LR, logistic regression; NB, Naïve Bayes; SVM, support vector machine.

**FIGURE 4:**
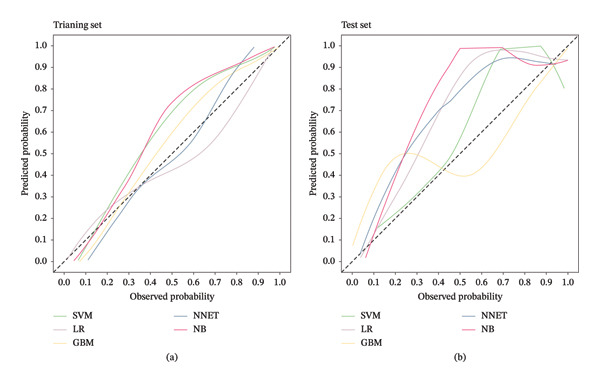
Calibration curves of the predictive models. (a) Training set; (b) test set. The horizontal axis represents the observed probability, and the vertical axis represents the predicted probability from the model. The black dashed line indicates the ideal curve, reflecting perfect agreement between the model’s predicted probability and the observed probability. Solid lines in different colors represent the calibration curves of different models, illustrating the variation trend of predicted probability with observed probability.

Figure 4 presents the calibration curves for the models. On the training set, except for the NB model, the calibration curves for the SVM, LR, GBM, and NNET models all aligned well with the ideal curve. Combined with the Hosmer–Lemeshow test results, the predicted and observed values showed no statistically significant difference for all five models (all *p* > 0.05). On the test set, the calibration curve for the NB model deviated significantly from the ideal curve, and the Hosmer–Lemeshow test indicated a significant difference between its predicted and observed values (*p* = 0.007). With the exception of the SVM model (*p* < 0.001), the predicted values of the LR, GBM, and NNET models remained consistent with the actual observed values (all *p* > 0.05). Further evaluation of the models′ calibration performance based on the Brier score showed that the LR model (training set = 0.095 and test set = 0.080) and the GBM model (training set = 0.053 and test set = 0.095) exhibited the best performance.

**FIGURE 5:**
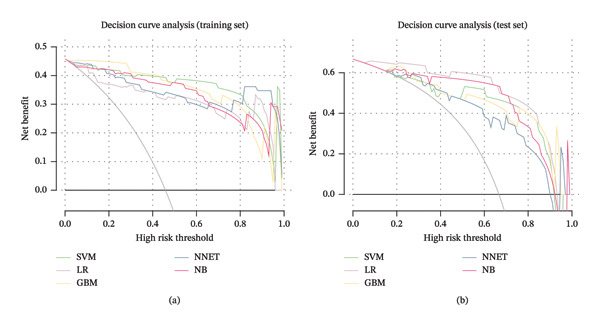
DCA curves of the predictive models. (a) Training set; (b) test set. The horizontal axis represents the high‐risk threshold, and the vertical axis represents the net clinical benefit. Curves in different colors illustrate the variation trend of the net clinical benefit of each model as the risk threshold changes. The black horizontal line indicates the net benefit of performing no clinical intervention for any patient, while the gray diagonal line represents the net benefit of providing clinical intervention for all patients.

DCA curves in Figure 5 revealed that the net clinical benefit of all models was consistently higher than the strategies of “treat none” and “treat all.” In the training set, the GBM and NNET models achieved the widest threshold probability ranges for net clinical benefit (0.02–0.98), while in the test set, the LR (0.06–0.93) and GBM (0.15–0.98) models showed the broadest ranges. Therefore, by comprehensively evaluating the discrimination, calibration, and clinical net benefit of each model in both the training and testing sets, the GBM model was identified as the optimal model for predicting BAV complicated with ascending aortic dilation.

### 3.6. Model Interpretation and Risk Stratification Based on GBM

**FIGURE 6:**
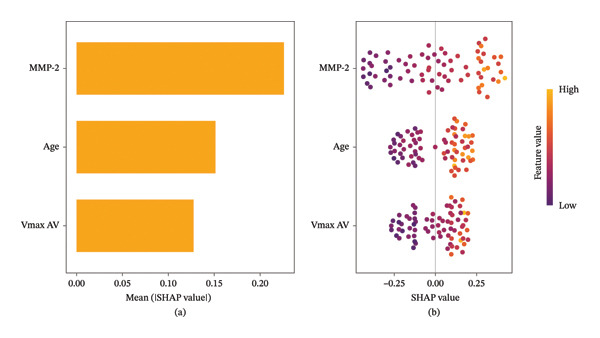
SHAP plots. (a) Bar plot of feature importance, the horizontal axis representing the average SHAP value, whose magnitude reflects the overall influence of the feature on the model’s predictions, and the vertical axis listing the individual features. The length of each bar indicates the ranking of feature importance. (b) Beeswarm plot, the horizontal axis denotes the SHAP value, while the vertical axis lists the features. The color of the points indicates the level of the feature value (higher values are shown in yellow, and lower values are shown in purple). The horizontal position of a point represents the direction of influence (points farther to the right indicate a stronger positive effect, and points farther to the left indicate a stronger negative effect). The density of point clustering reflects the distribution of samples.

This study employed SHAP analysis to provide a visual interpretation of the predictions generated by the GBM model. For global interpretation, the SHAP feature bar plot (Figure 6a) positioned plasma MMP‐2 (SHAP = 0.253) as the strongest predictor, followed by age (SHAP = 0.160) and Vmax AV (SHAP = 0.107). The SHAP swarm plot (Figure 6b) further uses color and point distribution to intuitively reflect the directional influence of each feature’s value on the prediction outcomes. The results indicate that elevated plasma MMP‐2 levels, increased age, and accelerated Vmax AV all raised the predicted value of the model.

**FIGURE 7:**
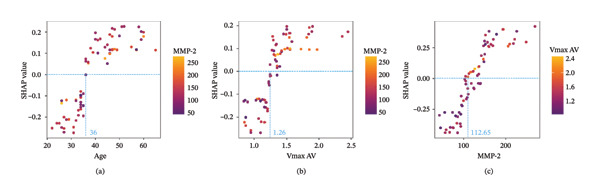
SHAP dependence plots. In (a) (age), (b) (Vmax AV), and (c) (MMP‐2), the horizontal axis represents the actual value of the feature, and the vertical axis represents the SHAP value (the contribution of each individual point), with each point representing one patient.

**FIGURE 8:**
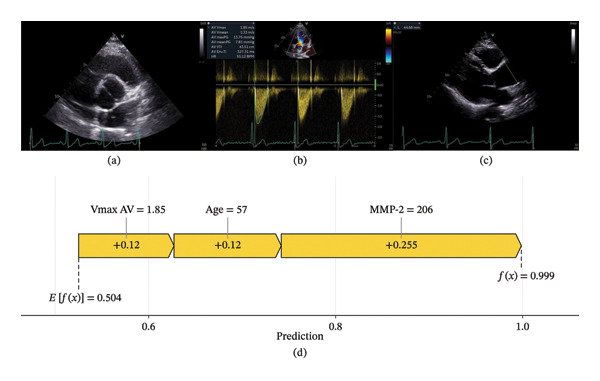
SHAP force plot of a BAV case. The data were derived from A 57‐year‐old patient with BAV (a), whose Vmax AV was 1.85 m/s (b) and plasma MMP‐2 concentration was 206 ng/mL. The SHAP force (d) shows the contribution of each feature when the GBM model assesses the risk of ascending aortic dilation in this patient. The *E* [*f*(*X*)] represents the baseline model risk (average predicted value of the entire sample). The final prediction *f*(*x*) for the current sample is obtained by summing the SHAP values of all features with the baseline risk. In this case, the baseline risk *E* [*f*(*x*)] = 0.504. After adding the SHAP values of plasma MMP‐2 (+0.255), age (+0.120), and Vmax AV (+0.120), the GBM model predicted the patient’s risk of ascending aortic dilation as *f*(*x*) = 99.9%. The actual measured diameter of the ascending aorta was 44.66 mm (c), indicating consistency between the model prediction and the clinical observation.

Furthermore, the SHAP dependence plot (Figure 7) illustrates the relationship between changes in a single feature’s value and the output of the model prediction. For BAV patients, when age > 36 years, Vmax AV > 1.26 m/s, and plasma MMP‐2 > 112.65 ng/mL, the GBM model predicts a significantly increased likelihood of ascending aortic dilation (SHAP > 0). The SHAP force plot (Figure 8) further demonstrates the independent contribution of each feature to the prediction outcome for specific samples, thereby visually presenting the model’s decision basis at the individual level.

Based on SHAP analysis and clinical practice, we performed a preliminary risk stratification for BAV patients. The specific stratification is as follows: (1) low risk: absence of all high‐risk features (i.e., plasma MMP‐2 < 113 ng/mL, age < 36 years, and Vmax AV < 1.3 m/s); (2) intermediate risk: presence of any one of the following combinations (a. plasma MMP‐2 > 113 ng/mL and age > 36 years; b. plasma MMP‐2 > 113 ng/mL and Vmax AV > 1.3 m/s; c. age > 36 years and Vmax AV > 1.3 m/s); (3) high risk: concurrent presence of all three high‐risk features (plasma MMP‐2 > 113 ng/mL, age > 36 years, and Vmax AV > 1.3 m/s).

## 4. Discussion

The diagnosis of ascending aortic dilation in patients with BAV is often an incidental finding on echocardiography. This frequent diagnostic delay contributes to a significantly elevated risk of serious complications, such as aortic dissection or rupture [6]. To gain further insight into the pathogenesis and progressive evolution of aortic dilation in BAV, numerous studies have focused on identifying circulating biomarkers capable of predicting structural alterations in the aortic wall, with a significant body of research centered on MMPs [19]. Based on previous studies [20, 21] and data availability, this study selected representative molecules of key functional MMP subtypes for plasma level measurement, including collagenase (MMP‐1), gelatinases (MMP‐2, MMP‐9), stromelysin (MMP‐3), and their endogenous tissue inhibitors (TIMP‐1, TIMP‐2, TIMP‐3, and TIMP‐4). The expression differences of these MMPs, TIMPs, and MMP/TIMP ratios in BAV patients with ascending aortic dilation were subsequently analyzed.

Previous studies by Wojnarski et al. [22] utilized unsupervised ML algorithms, while Micaelo et al. [23] employed random forests to investigate aortopathy associated with BAV. However, most existing predictive models are based on single‐modality data, such as radiomics or metabolomics. This study developed a multimodal approach by integrating circulating biomarkers (MMPs and TIMPs), echocardiographic parameters, and clinical data. Using LASSO‐LR, we identified age, Vmax AV, and plasma MMP‐2 as independent risk factors for ascending aortic dilation in BAV patients. Among the various models developed (SVM, LR, GBM, NNET, and NB), the GBM demonstrated superior overall diagnostic performance.

Compared with the traditional LR model, the GBM model, due to its inherent advantages in capturing the complex nonlinear relationships and interaction effects among variables, provides greater predictive flexibility for this study in exploring the complex pathogenesis and highly heterogeneous clinical phenotypes of the disease BAV. This advantage has been verified in multiple studies [13, 24]. This study utilized ROC curves, calibration curves, and DCA curves to evaluate the model’s discrimination, calibration, and clinical net benefit, respectively. Taking into account the overall performance of the model in both the training and test sets, the balance among different metrics, and its generalization ability, the GBM model demonstrated the best comprehensive diagnostic performance. However, the GBM model lacks the intuitive decision‐making process of traditional LR due to its complexity. To enhance interpretability while preserving the high performance of the GBM model, this study further applied SHAP analysis for post hoc model explanation. SHAP analysis quantifies the contribution of each feature to individual prediction outcomes, clearly identifying the variables that have the most significant impact on the model’s output. The SHAP analysis results indicate that elevated plasma MMP‐2 level was identified as the most important influencing factor for ascending aortic dilation in BAV patients, followed by increased age and elevated Vmax AV.

Aortic stiffening caused by age is a known outcome. This study found that increased age is an independent risk factor for ascending aortic dilation in BAV, a finding that agrees with the results of Thompson et al. [25] and Choi et al. [26]. Moreover, this study also found that Vmax AV, as an important echocardiographic indicator for assessing hemodynamics in BAV, is an independent risk factor for ascending aortic dilation, which is consistent with the research results of Irtyuga et al. [27]. Previous studies have demonstrated that an elevated maximum aortic valve velocity in patients with BAV is significantly associated with increased wall shear stress and vascular remodeling of the aorta [28]. Although patients with aortic stenosis were excluded from this study, the significantly abnormal leaflet fusion patterns in BAV can still lead to hemodynamic abnormalities within the aorta. Chronic exposure to these disturbed flow patterns may trigger medial layer remodeling (including endothelial damage and MMP activation), thereby accelerating the progression from aortic dilation to dissection [5].

It is known that the elastic function of the aortic wall in BAV patients is influenced by abnormal hemodynamics and potential genetic susceptibility, which collectively initiate matrix remodeling by modulating the MMP/TIMP balance [29, 30]. The findings of this study indicate that plasma MMP‐2 is an independent risk factor for ascending aortic dilation in BAV patients. This suggests that, compared to other MMPs, MMP‐2 may play a more dominant role in the early stages of aortic disease in BAV [31]. Previous studies [32] have shown that plasma MMP‐2 levels are significantly higher in BAV patients compared to healthy individuals and are positively correlated with endothelial dysfunction and ascending aortic diameter. As a gelatinase, MMP‐2 is directly involved in vascular remodeling in BAV patients by degrading elastin and collagen in the medial layer of the aortic wall and is considered a reliable marker for predicting ascending aortic dilation in BAV [33].

In this study, univariate LR analysis revealed that plasma TIMP‐2 levels and the MMP‐2/TIMP‐3 ratio are important influencing factors for BAV complicated with ascending aortic dilation. When the early stage of matrix remodeling in the aortic wall is highly active, the plasma level of MMP‐2 significantly increases. Concurrently, TIMP‐2, as a key physiological inhibitor of MMP‐2, also shows increased levels, indicating a compensatory protective response in the body by inhibiting protease activity [34, 35]. An elevated MMP‐2/TIMP‐3 ratio suggests that the metabolic balance of the aortic wall extracellular matrix remains skewed toward degradation at this stage, which may subsequently lead to progressive, irreversible aortic dilation. However, after controlling for intervariable influences through multivariate LR analysis, this study found that plasma TIMP‐2 levels and the MMP‐2/TIMP‐3 ratio were not independent influencing factors for BAV complicated with ascending aortic dilation. We speculate that a possible reason for this result is that the elevation of plasma TIMP‐2 in BAV patients largely represents a compensatory protective response of the body to the increase in MMP‐2, while the MMP‐2/TIMP‐3 ratio indirectly depends on MMP‐2 levels. This suggests that although TIMP‐2 and the MMP‐2/TIMP‐3 ratio play a significant role in the pathophysiological process of early aortic dilation, they may not be the direct and key driving factors for aortic dilation in BAV.

This study developed a risk prediction model for BAV combined with ascending aortic dilation based on GBM and applied SHAP analysis to interpret the contribution of each risk factor in individual patients, thereby enhancing the model’s interpretability and its applicability in clinical practice. Based on this interpretable risk prediction model, clinicians can perform preliminary risk stratification for BAV patients to support personalized decision management. For patients identified as low risk, regular annual echocardiographic follow‐up is recommended. For those in the intermediate‐risk category, in addition to regular annual echocardiographic follow‐up, initiation or intensification of cardiovascular risk factor management (such as hypertension and hyperlipidemia) is advised. For patients at high risk, it is recommended to shorten follow‐up intervals, enhance monitoring of aortic disease progression, and strengthen cardiovascular risk factor management. If necessary, individualized assessment of the timing of surgical intervention based on aortic diameter should be conducted to potentially reduce the risk of adverse cardiovascular events in patients with BAV.

This study also has the following limitations: First, the ascending aortic diameters of the patients in the study cohort did not meet the guideline‐recommended thresholds for surgical intervention, thus preventing a comparative analysis of MMPs and TIMPs at both tissue and plasma levels. Second, the sample size of this study was relatively small, and all data were derived from a single center. The AUC of the GBM model in the training set was higher than that in the test set, suggesting a potential risk of overfitting. In the future, we will perform external validation in larger scale, multicenter independent cohorts to evaluate the predictive performance of the model. Additionally, further exploration is warranted to assess the model’s performance differences across various BAV populations (such as those with different levels of valvular dysfunction and different valve fusion patterns) to enhance its clinical applicability.

## 5. Conclusions

In summary, this study identified age, Vmax AV, and plasma MMP‐2 as independent risk factors for ascending aortic dilation in patients with BAV. A GBM model was subsequently developed and validated as the optimal predictor for this condition. The integration of SHAP analysis successfully addressed the “black‐box” issue, significantly enhancing the model’s interpretability and clinical utility. It provides a scientific basis for clinicians to evaluate the progression and prognosis of aortopathy associated with BAV, supporting early intervention and personalized management.

## Funding

This study was financially supported by the Ningxia Natural Science Foundation (No. 2023AAC03635).

## Conflicts of Interest

The authors declare no conflicts of interest.

## Data Availability

The data that support the findings of this study are available from the corresponding author upon reasonable request.
